# Burden of *Clostridioides difficile* Infection and Risk Factors for Recurrences in an Italian Tertiary Care University Hospital: A Prospective Observational Study

**DOI:** 10.3390/antibiotics15010023

**Published:** 2025-12-25

**Authors:** Maria Chiara Gagliano, Giulio D’Agati, Alice Annalisa Medaglia, Luca Pipitò, Bianca Catania, Claudia Conti, Antonino Tuttolomondo, Angelo Baldassare Cefalù, Calogero Cammà, Nicola Scichilone, Anna Licata, Mario Barbagallo, Rita Immordino, Roberta Virruso, Giovanni Maurizio Giammanco, Antonio Cascio

**Affiliations:** 1 Department of Health Promotion, Mother and Child Care, Internal Medicine and Medical Specialties, University of Palermo, 90127 Palermo, Italy; mariachiara.gagliano@community.unipa.it (M.C.G.); giulio.dagati@community.unipa.it (G.D.); bianca.catania@community.unipa.it (B.C.); claudia.conti@community.unipa.it (C.C.); antonino.tuttolomondo@unipa.it (A.T.); abaldassare.cefalu@unipa.it (A.B.C.); calogero.camma@unipa.it (C.C.); nicola.scichilone@unipa.it (N.S.); anna.licata@unipa.it (A.L.); mario.barbagallo@unipa.it (M.B.); giovanni.giammanco@unipa.it (G.M.G.); 2 Infectious and Tropical Disease Unit, AOU Policlinico “P. Giaccone”, 90127 Palermo, Italy; alice.medaglia@policlinico.pa.it; 3 Antimicrobial Stewardship Team, AOU Policlinico “P. Giaccone”, 90127 Palermo, Italy; 4 Microbiology and Virology Unit, AOU Policlinico “P. Giaccone”, 90127 Palermo, Italy; rita.immordino@policlinico.pa.it (R.I.); roberta.virruso@policlinico.pa.it (R.V.)

**Keywords:** *Clostridium difficile*, *Clostridioides difficile*, CDI, diarrhea, hospital-acquired infection, pseudomembranous colitis, fidaxomicin, oral vancomycin, recurrence of CDI

## Abstract

**Background:**  *Clostridioides difficile* infection (CDI) remains a challenging condition, particularly in severe or recurrent cases. This study aimed to identify factors associated with recurrent CDI (rCDI), severe disease (defined by ZAR score or ESCMID criteria), death during CDI, and bloodstream infections (BSI) or candidemia within 8 weeks of CDI onset. **Methods:** We conducted a prospective study at an Italian university hospital that included all adult CDI cases diagnosed between November 2022 and December 2024. Statistical analyses were performed with IBM SPSS Statistics. A *p*-value < 0.05 was considered statistically significant in univariate analyses. For the multivariable analysis, we selected the variables that were statistically significant in the univariate analysis and considered the most clinically relevant. **Results:** A total of 161 CDI cases were identified. Recurrence occurred in 13%, higher than the 4% reported in a previous retrospective cohort at the same center (2013–2022). In univariate analysis, independent predictors of recurrent CDI (rCDI) were therapeutic regimens including oral vancomycin (*p* = 0.008; OR 6.17; 95% CI 1.36–27.97), peripheral vascular disease (PVD) (*p* < 0.001; OR 5.92; 95% CI 2.07–16.94), and dysphagia (*p* = 0.034; OR 4.61; 95% CI 1.25–17.07), whereas fidaxomicin use was associated with a protective effect (*p* = 0.016; OR 0.17; 95% CI 0.04–0.78). In multivariable analysis, oral vancomycin use (*p* = 0.008; OR 15.03) and peripheral vascular disease (*p* = 0.002; OR 7.27) remained independently associated with rCDI. Overall, 15 of 161 patients (9.3%) died during the CDI episode (either presenting CDI or rCDI), with all deaths directly attributable to CDI. Mortality during CDI was associated with age > 77 years (median value of the study population), transfer from a nursing home or long-term care facility within the previous 3 months, lymphoma, hematological malignancy, peripheral vascular disease, connective tissue disease, immobilization syndrome, dysphagia, elevated lactate levels (>1 mmol/L), septic shock, severe or severe-complicated CDI according to ESCMID criteria, severe-complicated CDI according to ESCMID criteria, leukocytosis (WBC > 15,000/mm^3^) during CDI, ZAR score ≥ 2, concomitant BSI, and concomitant pneumonia. During follow-up, 11 of 127 (8.7%) patients developed a BSI. BSI was associated with corticosteroid use and osteomyelitis. Only four patients developed candidemia due to *Candida albicans* during follow-up. **Conclusions:** Our study confirms that *Clostridioides difficile* infection remains a major clinical challenge, particularly due to its high recurrence rate and the burden of severe forms. The evidence strongly supports the preferential use of fidaxomicin, which should now be regarded as the standard of reference in clinical practice.

## 1. Introduction

*Clostridioides difficile* (CD) is an anaerobic, spore-forming Gram-positive bacillus responsible for toxin-mediated disruption of the intestinal epithelium, leading to diarrhea and potentially life-threatening complications such as fulminant colitis, sepsis, and toxic megacolon [1]. Although CD can be part of the gut microbiota, colonizing up to 50% of infants but <3% of healthy adults, it acts as an opportunistic pathogen, particularly in elderly or immunocompromised hospitalized patients [2]. Its virulence primarily derives from toxins A and B, which inactivate Rho GTPases, impair epithelial integrity, and trigger inflammation [1,2]. In Europe, CDI epidemiology remains incompletely characterized. ECDC estimates from 2018 to 2020 report hospital-associated CDI (HA-CDI) incidence rates of 2.58 cases per 10,000 patient-days and community-associated CDI (CA-CDI) rates of 1.35 per 1000 admissions [3]. Italian data are scarce: a 2016 surveillance study documented HA-CDI and CA-CDI rates of 2.26 and 0.5 per 10,000 patient-days [4], while Sicilian data from 2009 to 2019 identified 1139 cases, mostly in older adults (median age 73.2 years) and primarily from secondary or tertiary hospitals [5]. Mortality varies widely. Meta-analyses report 30-day mortality rates of 8–50% [6], whereas European multicenter data show a 13% rate [7]; an Italian multicenter study reported a 6.1% mortality rate in first-episode CDI [8]. Recurrent CDI (rCDI), defined as the reappearance of symptoms within 2–8 weeks, remains a major clinical challenge [9,10]. Risk factors include advanced age, recent hospitalization, proton pump inhibitor (PPI) exposure, HA-CDI, prior rCDI, and patient frailty [11]. European hospital incidence has remained stable at ~0.25–0.27 cases per 1000 discharges (2018–2020) [12], with recurrence rates ranging from 10% to 40% across studies [5,11,13,14]. Italian data are limited, reporting recurrence rates of 4% in one retrospective study and 21% in a multicenter cohort [8,15]. Strategies to prevent recurrence include bezlotoxumab, a monoclonal antibody targeting toxin B [16,17], and, in severe disease, intravenous therapy as an adjunct to oral regimens [18,19,20]. Beyond recurrence, CDI predisposes to secondary bloodstream infections (BSIs), particularly candidemia and infections due to enteric Gram-negative bacilli [21,22,23,24]. These events are likely facilitated by toxin-induced epithelial injury and increased intestinal permeability, promoting microbial translocation [25].

## 2. Objectives

Crucially, the study’s primary objective was to identify factors associated with rCDI, which guided the overall analytical approach.

The secondary objectives included:

- Characterizing CDI cases in terms of incidence, demographics, hospital ward distribution, clinical characteristics, severity, mortality, and treatment strategies.
- Assessing factors associated with severe CDI presentations.
- Examining predictors of death during CDI.
- Evaluating factors associated with bloodstream infections (bacteremia or candidemia) within 60 days of CDI onset.

## 3. Materials and Methods

We conducted a prospective observational study at the “Paolo Giaccone” University Hospital in Palermo, Italy, a 600-bed tertiary care center, between November 2022 and December 2024. All adult patients (aged 18 years or older) diagnosed with CDI were enrolled at the time of assessment at our center, regardless of whether they presented with a first episode (initial CDI) or a recurrence (rCDI), and each patient was included only once. Patients were excluded if the CDI diagnosis had been established at another hospital (even if subsequently managed at our center), if they were transferred to another facility during the CDI episode, or if the diagnosis was based solely on clinical suspicion without supportive microbiological evidence. Patients presenting with acute diarrhea (Bristol Stool Scale types 6–7) or other symptoms and signs suggestive of CDI, such as pseudomembranous colitis identified during endoscopy or ileus associated with radiological signs, were evaluated. Diagnosis was performed by analyzing diarrheic stool samples for glutamate dehydrogenase (GDH) and/or *Clostridioides difficile* toxins using enzyme immunoassays (ImmunoCard^®^ assay, Meridian Bioscience, Cincinnati, OH, USA) or PCR-based methods (Xpert^®^ *C. difficile* BT assay, Cepheid, Sunnyvale, CA, USA). For community-onset diarrhea only, the Seegene Allplex™ GI-Bacteria (I) assay (Seegene, Seoul, Republic of Korea) was used as the initial diagnostic method to identify the causative pathogen. In cases with high clinical suspicion of CDI, the diagnostic algorithm recommended by the ESCMID guidelines was applied [10]. Specifically, highly sensitive GDH and Tox A/B enzyme immunoassays (EIA) were performed: a CDI diagnosis was confirmed if both tests were positive; if results were discordant, a nucleic acid amplification test (NAAT) was performed; if both tests were negative, CDI was ruled out. Ribotype 027 was assessed by NAAT performed concurrently with toxin detection. All testing was conducted by the Microbiology Unit in response to a formal diagnostic request. No routine screening for CD colonization was performed. Each CD-positive sample was reported to our antimicrobial stewardship team by the microbiology unit. New CDI cases were initially reported directly by the Microbiology Unit. Trained infectious disease (ID) personnel then visited the ward to collect clinical data from the patient and the electronic medical record, entering information into a predefined standardized case report form that included known risk factors for recurrence, CDI classification, informed consent, and patient contact details for follow-up within 8 weeks by phone or in-person. All data were subsequently entered into a centralized database. Upon the ward team’s request, the ID specialist assessed the patient, provided CDI management recommendations, and documented any additional interventions.

Fidaxomicin was prescribed only after ID consultation. As recommended by our hospital, fidaxomicin was prescribed in all cases where the doctor deemed the infection more severe or the risk of recurrence was considered higher.

During the 8-week follow-up period, rCDI, other infections, and antibiotic exposure were monitored. For patients discharged before the end of the follow-up period, the patient was contacted by phone and interviewed regarding the onset of new clinical symptoms or potential concomitant conditions.

For each episode of CDI, the following data were collected:

- Epidemiological data (age, sex, nationality);
- Date and hospital ward where CDI onset occurred, and length of hospital stay (LOS);
- Presence of known CDI risk factors at the time of diagnosis, including chronic use of antacids or PPIs, infections and/or antibiotic therapy within the previous 3 months, immunosuppression, prior hospitalization or transfer from another healthcare facility within the preceding 3 months, abdominal surgery within 3 months before CDI onset, inflammatory bowel disease, chronic kidney disease, and liver cirrhosis;
- Comorbidities;
- Concurrent infections and associated antimicrobial therapies;
- CDI-related characteristics, including primary or secondary episode, healthcare-associated or community-acquired form, clinical presentation, laboratory findings (leukocyte count, C-reactive protein (CRP), procalcitonin (PCT), serum creatinine, serum albumin), diagnostic test results, severity classification according to ZAR score (according to Zar et al., 2007 [26] a validated clinical tool derived from hospitalized patient cohorts that incorporates age, temperature, leukocyte count, serum albumin, and comorbidities to stratify patients according to the risk of severe disease and CDI-related mortality) or ESCMID criteria, and treatment administered;
- All-cause mortality during the CDI episode, occurrence of bacteremia or candidemia within 60 days of CDI onset, and CDI recurrence.

In cases of recurrent rCDI, the same data were collected, and follow-up was extended by 8 weeks. Recurrent CDI (rCDI) was defined according to the 2021 ESCMID guidelines [10] as the reappearance of symptoms within 8 weeks after resolution of the initial episode. In our study, the term secondary CDI (sCDI) was used to denote a new CDI episode occurring in the same patient after the 8-week window, outside the temporal definition of recurrence. All definitions of CDI, rCDI, severe CDI, healthcare-associated CDI, and community-acquired CDI, as well as the diagnostic methods, were based on the 2021 guidelines of the European Society of Clinical Microbiology and Infectious Diseases (ESCMID) [10] and the Infectious Disease Society of America [9]. In the few cases where the diagnosis did not fully align with current guideline recommendations, the attending physician made the final treatment decision.

Continuous variables were described using the median, mean ± standard deviation (SD), interquartile range (IQR), and 95% confidence intervals (CI). Categorical variables were summarized as absolute and relative frequencies (%). Statistical analyses were performed with IBM SPSS Statistics for Windows, version 26 (IBM Corp., Armonk, NY, USA). Univariate analyses were performed to calculate crude odds ratios (ORs) and corresponding 95% confidence intervals (CIs) for each outcome and examined factor. Statistical significance was set at a *p*-value < 0.05. For the multivariable analysis, we selected variables that showed a statistically significant association in the univariate analysis and were considered clinically meaningful based on their biological plausibility and relevance to CDI outcomes.

## 4. Results

### 4.1. Distribution, Incidence, and Patient Features

**Figure 1:**
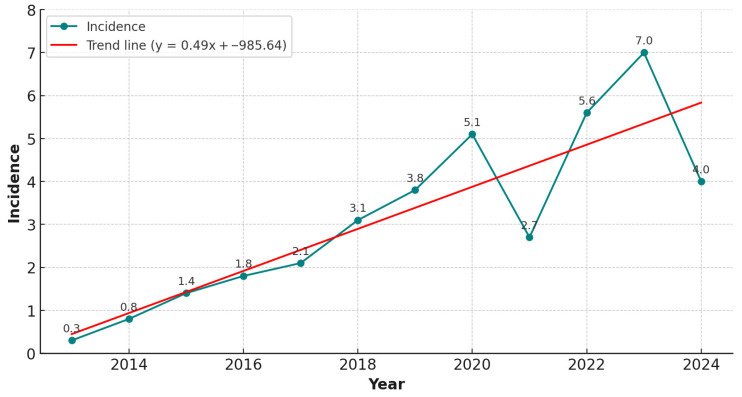
Incidence of CDI cases per 10,000 patient-days from 2013 to 2024, based on data from a previously conducted retrospective observational study (2013–2022) [15] and the present prospective observational study (2022–2024).

During the study period, 162 patients were reported to have experienced a CDI episode, and 161 were enrolled. The incidence rates were 7.0 per 10,000 patient-days in 2023 and 4.0 per 10,000 patient-days in 2024 (see Figure 1).

**Figure 2:**
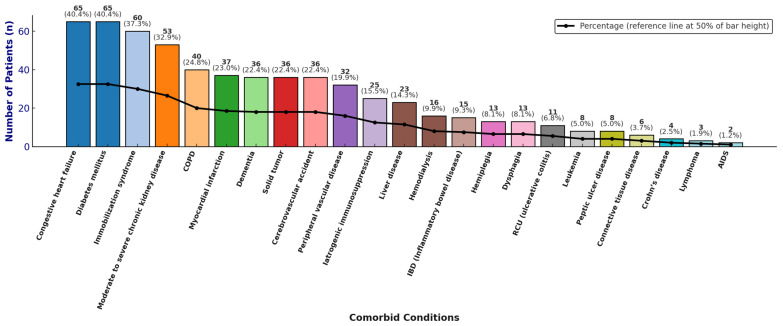
Distribution of comorbidities, absolute and relative frequencies.

A total of 127/161 patients (78.9%) completed the 8-week follow-up from the onset of the CDI episode. Females and males accounted for 81/161 (50.3%) and 80/161 (49.7%) of cases, respectively, with a male-to-female ratio of 1.01. The median LOS was 26 days. A total of 159/161 patients (98.8%) were of Italian nationality and Caucasian ethnicity, while 2/161 patients (1.2%) were from Africa. The majority of patients were elderly at the time of diagnosis: 117 out of 161 (72.7%) were aged 65 years or older, with a median age of 72 years (mean 69 ± 14 SD; IQR: 63–78). The wards with the highest proportions of CDI cases were internal medicine (40/161, 24.8%), gastroenterology (25/161, 15.5%), and infectious and tropical diseases (21/161, 13.0%; see Supplemental Figure S1). Many patients presented with multiple comorbidities; the mean Charlson comorbidity index (CCI) was 4, with an age-adjusted mean of 7. The most prevalent comorbidities were congestive heart failure and diabetes mellitus (both 65/161, 40.4%) (see Figure 2).

### 4.2. Concomitant Infection

Concomitant infections were identified in 132 of 161 patients (82.0%). The most frequent were pneumonia (55/161, 34.2%), urinary tract infections (UTIs) (39/161, 24.2%), and BSIs (23/161, 14.3%). These were followed by intra-abdominal infections (IAIs) (18/161, 11.2%), acute bacterial skin and skin structure infections (ABSSSIs) (7/161, 4.3%), osteomyelitis (3/161, 1.9%), infective endocarditis (3/161, 1.9%), and central nervous system infections (CNSIs) (2/161, 1.2%). The isolated microorganisms were Gram-negative in 50 of 161 cases (31.1%) and Gram-positive in 32 of 161 cases (19.9%). Among bacterial isolates, *Klebsiella pneumoniae* was the most frequent (28/161, 17.4%), followed by *Escherichia coli* (13/161, 8.1%) and *Enterococcus* spp. (11/161, 6.8%). Viral infections were documented in 14 of 161 cases (8.7%), while candidemia occurred in 7 of 161 cases (4.3%). The most frequently administered antibiotics were beta-lactams, including penicillins (58/161, 36.0%), cephalosporins (53/161, 32.9%), and carbapenems (34/161, 21.1%), in addition, antifungal therapy was administered in 8/161 cases (5.0%) and antiviral therapy in 2/161 cases (1.2%), see Supplemental Figure S2.

### 4.3. Presentation of CDI and Management

The majority of CDI cases occurred as initial episodes (iCDI), accounting for 152 of 161 cases (94.4%). Secondary episodes (sCDI) were documented in 5/161 cases (3.1%), while rCDI, each representing the first episode captured within the study period, was observed in 4/161 cases (2.5%). Most infections were classified as HA-CDI, accounting for 143/161 cases (88.8%), while CA-CDI accounted for 18/161 cases (11.2%). Diarrhea was the predominant symptom across all CDI episodes and was associated with abdominal pain in 46 of 161 patients (28.6%) and/or fever (body temperature ≥ 38.5 °C) in 13/161 patients (8.1%). Vomiting was reported in 6 of 161 patients (3.7%). Septic shock represented the most frequent complication (9/161, 5.6%), followed by paralytic ileus (5/161, 3.1%). 53/161 CDI episodes (32.9%) were classified as severe based on a ZAR score of ≥2. Based on ESCMID criteria, 45/161 cases (27.9%) were categorized as severe, and 20/161 (12.4%) as severe-complicated.

Among laboratory parameters, leukocytosis (WBC ≥ 15,000/mm^3^) was observed in 36/161 patients (22.4%), while increased serum creatinine levels (>50% above baseline) were documented in 10/161 patients (6.2%). Hypoalbuminemia (<2.5 mg/dL) occurred in 17/161 cases (10.5%). Electrolyte abnormalities were frequent, with hyponatremia (<135 mmol/L) detected in 134/161 patients (83.2%) and hypokalemia (<3.5 mmol/L) in 106/161 patients (65.8%). Elevated serum lactate levels (>1 mmol/L) were reported in 26 of 106 patients tested (24.5%). Median, mean ± SD, and IQR values are reported in Table 1.

The CD-GDH assay was positive in 155 of 156 tested cases (99.3%), whereas the toxin enzyme immunoassay yielded positive results in 115 of 140 cases (82.1%). Nucleic acid amplification testing (NAAT) for toxin B was performed in 62 patients, of whom 56 (90.3%) tested positive; the binary toxin gene was detected in 12/62 cases (19.3%). Ribotype 027 testing was performed in 42 patients, with no positive isolates identified.

**Figure 3:**
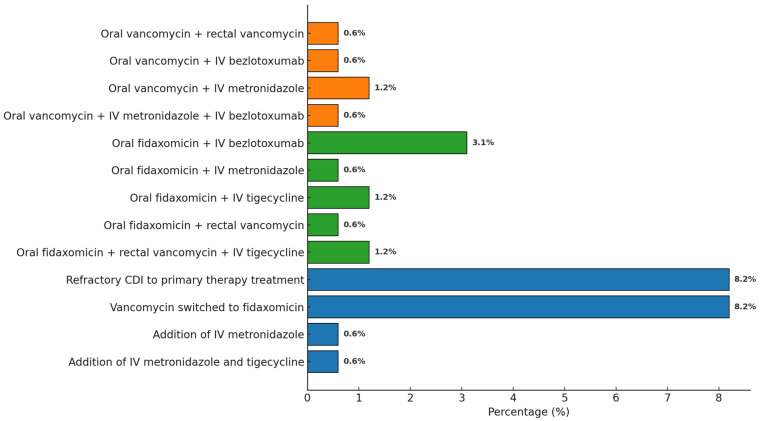
Distribution of antibiotic combination therapies administered for CDI.

Two patients did not receive anti-CDI therapy due to death occurring within the first hours after diagnosis. Initial treatment with oral vancomycin alone and oral fidaxomicin alone was administered in 89 out of 159 cases (56.0%) and 56 out of 159 cases (35.2%), respectively. Intravenous bezlotoxumab was added in 7/159 cases (4.4%) based on the assessed risk of rCDI. Intravenous metronidazole or intravenous tigecycline was added in 4/159 cases (2.5%) due to severe infection. Rectal vancomycin was administered in 4/159 cases (2.5%). Thirteen of 89 cases (14.6%) were refractory to initial vancomycin therapy. In these cases, vancomycin was switched to fidaxomicin. Intravenous metronidazole was added in 1/159 case (0.6%), and both intravenous metronidazole and intravenous tigecycline were added in another case of 161 (0.6%) (see Figure 3).

Of the 126 patients receiving systemic antibiotics for concomitant infections, treatment could be discontinued in 73 cases, whereas 53 patients (42.1%) remained on antibiotic therapy during the CDI episode. The concomitant infections in these 53 patients are depicted in Supplemental Figure S3. Among these, five experienced a recurrence, all of whom had been treated with vancomycin.

Of the 135 patients on chronic PPI therapy, treatment was discontinued in 16 cases (11.9%). Despite being contraindicated, antidiarrheal agents were administered in 8 out of 161 patients (5.0%). Infectious disease consultation was requested in 134 out of 161 cases (83.2%).

### 4.4. CDI and Outcomes

#### 4.4.1. Recurrent CDI

rCDI was observed in 17 of 127 (13.4%) patients during follow-up, with a median time to recurrence of 32 days (mean 34 ± 10 SD, IQR 27–44) from the presenting CDI episode. The cohort with rCDI included 6 men and 11 women (M:F ratio 1:1.8), of whom 11/17 (64.7%) were aged ≥65 years. The mean CCI was 5 (age-adjusted CCI: 9). According to ESCMID criteria, 10/17 (58.8%) presented with severe or severe-complicated disease. During follow-up, 3/17 (17.6%) developed a bloodstream infection (BSI), with no cases of candidemia, and 2/17 (11.8%) experienced a second recurrence. Among the 110 patients without rCDI at the end of follow-up, 57 were men, and 55 were women (M:F ratio 1.04:1), and 78/110 (70.9%) were aged ≥65 years. The mean CCI was 4 (age-adjusted CCI: 7). Severe or severe-complicated disease (ESCMID definition) was observed in 38/110 (34.5%). During follow-up, 8/110 (7.3%) developed BSI and 4/110 (3.64%) developed candidemia.

Oral vancomycin, dysphagia, and PVD were associated with a higher rCDI incidence at univariate analysis, whereas fidaxomicin was associated with a lower rCDI incidence (Table 2). In the multivariable analysis, oral vancomycin use (*p* = 0.008; OR 15.03; 95% CI 2.03–111.25) and peripheral vascular disease (*p* = 0.002; OR 7.27; 95% CI 2.05–25.78) were independent predictors of rCDI (Table 2). Among those with rCDI, 11/17 (64.7%) were females, and 6/17 (35.3%) were males, with median ages of 76 and 62 years, respectively. The median CCI was 5 (age-adjusted median: 8). Of 17 patients, 15 (88.2%) had a concomitant infection requiring systemic antibiotic therapy. In 2/17 (11.8%) cases, the presenting CDI episode was itself a recurrence. Most recurrences were HA-CDI (16/17, 94.1%). According to the ZAR score, 8/17 (47.1%) rCDI episodes were classified as severe. Based on ESCMID criteria, 7/17 (41.2%) were severe, and 3/17 (17.6%) were severe-complicated. Two of seventeen patients (11.8%) were refractory to first-line antibiotic treatment. Fidaxomicin was administered in 15/17 (88.2%) cases, while oral vancomycin was used in 2/17 (11.8%). Intravenous bezlotoxumab was added in 9/17 (52.9%) cases. Four out of seventeen patients (23.5%) died during the rCDI episode, while two/seventeen patients (11.8%) experienced a second recurrence. Univariate and multivariable analysis results are reported in Table 2.

#### 4.4.2. Death During CDI

Among the 161 enrolled patients, 15 died during the CDI episode; 5 died after CDI resolution while still hospitalized; 12 died after CDI resolution following discharge during the follow-up period; and 2 were lost to follow-up for reasons other than death. The remaining 127 patients effectively completed the follow-up.

Fifteen of 161 patients (9.3%) died during the CDI episode (presenting CDI or rCDI), with all deaths directly attributable to CD. Among them, 8/15 (53.3%) were females and 7/15 (46.7%) were males, with a median age of 77 and 60 years, respectively. Results of the univariate analysis for death during CDI are reported in Table 3.

In the multivariable analysis, the following variables were included: age > 77 years, transfer from nursing home/LTCF within the previous 3 months, lymphoma, hematological malignancy, severe or severe-complicated disease according to ESCMID criteria, severe-complicated disease according to ESCMID criteria, ZAR score ≥ 2, and septic shock.

Lymphoma (*p* = 0.046; OR 22.86; 95% CI 1.05–497.15) and septic shock (*p* = 0.001; OR 34.94; 95% CI 4.18–291.52) were identified as statistically significant factors associated with mortality during CDI.

#### 4.4.3. BSI

During follow-up, 11 of 127 (8.7%) patients developed a BSI. These were more frequently caused by Gram-positive bacteria (either monomicrobial or polymicrobial), with the most commonly isolated organisms being *Enterococcus faecalis* (25%), *Enterococcus faecium* (8%), and *Staphylococcus aureus* (8%). Among Gram-negative BSIs (mostly polymicrobial), the most frequent pathogens were *Klebsiella pneumoniae* (16%), *Proteus mirabilis* (16%), *Pseudomonas aeruginosa* (8%), *Acinetobacter baumannii* (8%), *Escherichia coli* (8%), and *Morganella morganii* (8%). A total of 4 of 127 (3.1%) developed a *Candida albicans* candidemia. Statistically significant predictors of bacterial BSI during follow-up included immunosuppression due to corticosteroid therapy (*p* = 0.039; OR, 4.47; 95% CI, 1.18–16.87) and osteomyelitis (*p* = 0.024; OR, 16.44; 95% CI, 2.07–130.62), both of which were confirmed in the multivariable analysis. Complicated diabetes mellitus was also associated with bacterial BSI at follow-up (*p* = 0.039; OR, 4.47; 95% CI, 1.18–16.87), although this association was not confirmed in multivariable analysis. Only 4 patients developed candidemia due to *Candida albicans* during follow-up.

## 5. Discussion

We observed incidence rates of 7.04 and 4.04 cases per 10,000 patient-days in 2023 and 2024, respectively, which were notably higher than those reported by the ECDC for 2018 (2.79), 2019 (2.02), and 2020 (2.58 cases per 10,000 patient-days). Although management protocols and diagnostic procedures remained unchanged, the reduction in incidence between 2023 and 2024 may, speculatively, reflect improved adherence to contact isolation practices, although this trend requires confirmation through longer-term observation. Our study confirms an increase in CDI incidence compared with the 2018–2020 ECDC report [3]. These findings align with European trends, in which recurrence is one of the most clinically significant complications of CDI and may reach 40% in some cohorts [11,12,13,14]. A retrospective study conducted at our center, covering the period from 2013 to October 2022, reported a rCDI incidence of 4% [15]. In contrast, our prospective study shows a marked increase in rCDI incidence, reaching 13% among CDI cases evaluated between November 2022 and December 2024. This significant rise may partly be attributable to the differences in study design (retrospective vs. prospective) and to previously underreported CDI cases, potentially due to a lack of discharge coding in earlier years.

In our cohort, the use of oral vancomycin as first-line therapy was associated with a higher incidence of rCDI. Instead, oral fidaxomicin use emerged as a statistically significant protective factor, reinforcing current IDSA and ESCMID recommendations that advocate fidaxomicin as first-line therapy [9,10]. These findings are consistent with earlier large-scale comparative trials evaluating fidaxomicin versus vancomycin in the treatment of CDI, such as the OPT-80-003 [27] (conducted between 2006 and 2008) and OPT-80-004 [28] (conducted between 2007 and 2009) studies. Both were phase III, multicenter, double-blind, randomized controlled trials. These studies demonstrated the non-inferiority of fidaxomicin compared to vancomycin in achieving clinical cure, along with a significantly lower recurrence rate of CDI. Also, a recent 2024 meta-analysis demonstrated that fidaxomicin was superior to vancomycin in reducing the risk of rCDI [29]. Our study also identified PVD and dysphagia as independent factors associated with rCDI. While some studies have reported cardiovascular disease, in general, as a risk factor for rCDI, they have not isolated individual components such as PVD. For instance, a Romanian study [30] from 2020 found that the presence of cardiovascular comorbidities or the use of cardiovascular medications increased the risk of rCDI. The association between dysphagia and rCDI may be explained by several factors, including an increased risk of malnutrition and dehydration, greater exposure to nosocomial pathogens and medical interventions, and alterations in the gut microbiota, all of which can facilitate the persistence or recurrence of rCDI. Although some studies have broadly linked cardiovascular disease to recurrence [30], few have analyzed the specific contribution of PVD. This condition may reflect endothelial dysfunction and impaired tissue perfusion, potentially compromising intestinal mucosal healing and favoring the persistence of toxins or the recolonization by toxigenic strains.

The association between dysphagia and rCDI may be explained by multiple factors. Dysphagia is a clinical marker of frailty and is often accompanied by malnutrition, dehydration, and an increased risk of aspiration pneumonia. These patients are also more frequently exposed to broad-spectrum antibiotics and nosocomial pathogens, and they undergo more frequent medical interventions. Altogether, these elements contribute to dysbiosis and impaired intestinal barrier integrity, thereby facilitating the persistence or recurrence of CDI.

Elevated creatinine levels similarly aligned with known risk factors for recurrent and severe CDI [7,8]. Renal impairment and systemic inflammation profoundly alter the gut microbiota and mucosal immune responses, thus increasing the likelihood of recurrence after treatment.

Overall, our findings suggest that recurrence risk results from an interplay between therapeutic choices and patient vulnerability. The presence of PVD, dysphagia, and renal impairment identifies subgroups at substantially increased risk of rCDI, for whom more aggressive preventive strategies, such as early fidaxomicin use and adjunctive bezlotoxumab, may be particularly appropriate.

These observations underscore the importance of a personalized therapeutic approach, based not only on severity classification but also on individualized risk profiling and overall patient frailty. Integrating our real-world data with existing international evidence reinforces the notion that systematic use of fidaxomicin and careful selection of high-risk patients can substantially reduce recurrent CDI.

Severe forms of CDI, as defined by the ZAR score or the ESCMID severity criteria, are widely recognized as factors associated with increased risk of death during CDI, a finding also confirmed in our study. A retrospective study published in 2014 reported that patients classified as having severe CDI according to the ZAR score exhibited significantly higher mortality rates compared to those with non-severe disease [31]. The association between ESCMID-defined severe CDI and increased mortality is also supported by the ESCMID guidelines themselves [10] and a European multicenter study conducted between 2011 and 2019 [32].

Our study demonstrated a statistically significant concordance between the ZAR score and ESCMID criteria in identifying severe cases of CDI. However, the ESCMID criteria classified more cases as severe (65 cases according to ESCMID vs. 53 according to the ZAR score). Both scoring systems were useful for predicting mortality during CDI, although the ZAR score showed greater statistical significance (*p* = 0.003 for ZAR vs. *p* = 0.029 for ESCMID). Several studies have compared different severity assessment tools for CDI in predicting mortality risk. Katzer et al. and Gomez-Simmonds et al. both reported that the ZAR score outperformed the ESCMID criteria in predicting 30-day mortality among patients with CDI. Although the ESCMID criteria remain valid, their prognostic performance appears to be inferior in terms of discriminatory capacity [31,33]. In our analysis, advanced age (>72 years), recent hospitalization, and baseline serum creatinine levels > 1.05 mg/dL were independent predictors of severity, as determined by the ZAR score. Advanced age is one of the most commonly recognized risk factors for CDI onset, severe disease, and recurrence. Similarly, elevated serum creatinine is widely acknowledged as a predictor of disease severity in CDI [7,8]. Katzer et al., in a single-center study conducted in the United Kingdom, also identified hospitalization within the previous three months as a factor associated with both severe CDI (as defined by the ZAR score) and 30-day mortality [33]. Abdominal pain was confirmed in our multivariable analysis as a predictor of severe disease according to ESCMID criteria, despite not being included among the formal ESCMID severity indicators. Khanafer et al. also reported abdominal pain as an independent variable associated with severe forms of CDI in their multivariable model [34].

Risk factors for BSI during or within 8 weeks of CDI onset in our study included corticosteroid-induced immunosuppression, osteomyelitis, and complicated diabetes mellitus. Giuliano et al. identified several risk factors for BSI associated with CDI, including prior antibiotic therapy, severe or severe-complicated CDI as defined by ESCMID criteria, and comorbid conditions such as dementia, neurological disorders, immobilization syndrome, dehydration, and hypoalbuminemia [23]. Ulrich et al. reported significant risk factors such as chronic kidney disease, malignancy, diabetes mellitus, liver failure, prolonged hospitalization, immunosuppressive therapy, use of invasive devices, and broad-spectrum antibiotics administered before or during CDI [24].

### Study Limitations

This study has several limitations that should be acknowledged.

First, the sample size was relatively small, particularly in the rCDI group, which resulted in limited statistical power. As a consequence, some odds ratios and confidence intervals appeared unusually wide or unstable, reflecting sparse data in several subgroups rather than computational inaccuracies. Likewise, the restricted number of events constrained the multivariable modeling strategy: only a limited set of covariates could be included to avoid overfitting and model instability, and other well-established confounders could not be incorporated.

Second, among the 161 enrolled patients, a small subset (*n* = 4) was evaluated during a recurrent episode of CDI rather than an initial or secondary episode. Although these cases were retained to preserve the real-world nature of the cohort, this heterogeneity may have introduced some misclassification bias.

Third, an infectious disease consultation was not consistently requested for all patients. Since this evaluation was required for prescribing fidaxomicin, variations in consultation practices may have influenced therapeutic choices and, consequently, clinical outcomes.

Despite these limitations, this study provides valuable prospective data on CDI and rCDI in a real-world tertiary-care setting, contributing relevant insights into epidemiology, clinical features, and treatment patterns.

## 6. Conclusions

The adoption of a prospective design with active, systematic follow-up enhanced the reliability of our data and enabled the identification of additional risk factors beyond those traditionally reported, such as peripheral vascular disease, dysphagia, and elevated serum creatinine. These results demonstrate how a structured methodological approach can reveal clinically significant determinants with important implications for both clinical practice and epidemiological surveillance. Our study confirms that *Clostridioides difficile* infection remains a major clinical challenge, particularly due to its high recurrence rate and the burden of severe forms. The evidence strongly supports the preferential use of fidaxomicin, which should now be regarded as the standard of reference in clinical practice. Its protective effect against recurrence, consistently observed in our cohort and in international trials, highlights the need for a paradigm shift in CDI treatment, moving away from vancomycin as first-line therapy. At the same time, the early and systematic evaluation of risk factors, including peripheral vascular disease, dysphagia, and renal impairment, allows for a more tailored therapeutic approach, ensuring better outcomes in high-risk patients. Taken together, these findings support a management strategy that integrates routine adoption of fidaxomicin with prospective surveillance and individualized risk stratification. Such an approach has the potential to substantially reduce recurrence, complications, and mortality, and ultimately reduce the overall CDI burden.

## Figures and Tables

**Table 1:** Laboratory parameters at CDI presentation.

Laboratory Parameters	Median (Interquartile Range) Mean ± SD
WBC (N°/mmc)	9990 (6820–14,040) 12,719 ± 10,333
Basal creatininemia (mg/dL)	1.05 (0.81–2.00) 1.77 ± 1.66
Creatininemia during CDI	0.96 (0.68–1.68) 1.56 ± 1.54
CRP (mg/L)	48.8 (18.8–117) 81.6 ± 84.7
PCT (mcg/L)	0.511 (0.201–1.487) 5.268 ± 14.672

Abbreviations: CDI (*Clostridioides difficile* infection), CRP (C-reactive protein), PCT (procalcitonin), SD (standard deviation), and WBC (white blood cell).

**Table 2:** Univariate and multivariable statistical analysis of factors associated with rCDI. Percentages were calculated based on a total of 127 patients who completed follow-up according to our study protocol.

Variables	rCDI (*n* = 17)	Non rCDI (*n* = 110)	OR	CI	*p*-Value
Univariate analysis					
Oral vancomycin	15 (11.8%)	79 (62.1%)	6.17	1.36–27.97	0.008
Rectal vancomycin	2 (1.6%)	2 (1.6%)	9.47	1.24–72.15	0.056
Fidaxomicin	2 (1.6%)	63 (49.6%)	0.17	0.04–0.78	0.016
rCDI (presenting CDI)	2 (1.6%)	2 (1.6%)	9.47	1.24–72.15	0.056
Dysphagia	4 (3.1%)	9 (7.1%)	4.61	1.25–17.07	0.034
Increased creatininemia	9 (7.1%)	43 (33.8%)	2.64	0.96–7.31	0.054
Peripheral vascular disease	9 (7.1%)	23 (18.1%)	5.92	2.07–16.94	<0.001
Multivariable analysis					
Oral vancomycin	15 (11.8%)	79 (62.1%)	15.03	2.03–111.25	0.008
Dysphagia	4 (3.1%)	9 (7.1%)	2.18	0.35–13.60	0.403
Peripheral vascular disease	9 (7.1%)	23 (18.1%)	7.27	2.05–25.78	0.002
Age ≥ 65 years	11 (8.7%)	106 (83.5%)	0.60	0.17–2.10	0.423
rCDI (presenting CDI)	2 (1.6%)	2 (1.6%)	6.50	0.69–61.12	0.102
ZAR score ≥ 2	8 (6.3%)	45 (35.4%)	1.56	0.45–5.43	0.481
Recent hospitalization *	9 (7.1%)	77 (60.6%)	1.30	0.29–5.76	0.729
Transfer from nursing home/LTCF	4 (3.1%)	18 (14.2%)	1.84	0.35–9.97	0.475
Recent antibiotic use *	8 (6.3%)	73 (57.5%)	0.65	0.14–2.95	0.580

* within the previous 3 months. Abbreviations: CDI (*Clostridioides difficile* infection), CI (confidence interval), LTCF (long-term care facility), OR (Odds ratio), rCDI (recurrent CDI).

**Table 3:** Univariate and statistical analysis of factors associated with death during CDI. Percentages were calculated based on a total of 161 enrolled patients.

Variables	Death During CDI (*n* = 15)	Survivors (*n* = 146)	OR	95% CI	*p*-Value
Univariate analysis					
Age > 77 years *	12 (7.5%)	68 (42.2%)	1.58	0.54–4.68	0.014
Transfer from nursing home/LTCF *	6 (3.7%)	16 (9.9%)	5.42	1.70–17.21	0.002
Lymphoma	2 (1.2%)	1 (0.6%)	22.31	1.89–262.84	0.023
Hematological malignancy	3 (1.9%)	6 (3.7%)	5.83	1.29–26.30	0.039
Peripheral vascular disease	6 (3.7%)	26 (16.1%)	3.08	1.01–9.40	0.040
Connective tissue disease	2 (1.2%)	1 (0.6%)	22.31	1.89–262.84	0.023
Immobilization syndrome	11 (6.8%)	49 (30.4%)	5.44	1.65–17.98	0.002
Dysphagia	4 (2.5%)	9 (5.6%)	5.53	1.47–20.89	0.021
Increased lactates (>1 mmol/L)	6 (3.7%)	20 (12.4%)	3.70	1.08–12.72	0.029
CRP > 48.8 mg/L * during CDI	10 (6.2%)	67 (41.6%)	3.53	0.93–13.39	0.050
Septic shock	7 (4.3%)	2 (1.2%)	63.00	11.22–353.66	<0.001
Severe or severe-complicated CDI (ESCMID criteria)	10 (6.2%)	55 (34.2%)	3.31	1.07–10.12	0.029
Severe-complicated CDI (ESCMID criteria)	7 (4.3%)	13 (8.1%)	8.95	2.80–28.65	<0.001
WBC > 15,000/mm^3^ during CDI	7 (4.3%)	29 (18.0%)	3.53	1.18–10.53	0.018
ZAR score ≥ 2	10 (6.2%)	43 (26.7%)	4.79	1.55–14.84	0.003
Contextual BSI	5 (3.1%)	18 (11.2%)	3.56	1.09–11.59	0.027
Contextual PNA	10 (6.2%)	45 (28.0%)	4.49	1.45–13.89	0.005
Multivariable analysis					
Age > 77 years *	12 (7.5%)	68 (42.2%)	0.58	0.13–2.60	0.475
Transfer from nursing home/LTCF *	6 (3.7%)	16 (9.9%)	4.13	0.75–22.86	0.104
Lymphoma	2 (1.2%)	1 (0.6%)	22.87	1.05–497.15	0.046
Hematological malignancy	3 (1.9%)	6 (3.7%)	6.54	0.58–73.87	0.129
Severe or severe-complicated CDI (ESCMID criteria)	10 (6.2%)	55 (34.2%)	0.53	0.03–8.11	0.651
Severe-complicated CDI (ESCMID criteria)	7 (4.3%)	13 (8.1%)	4.14	0.53–32.48	0.176
ZAR score ≥ 2	10 (6.2%)	43 (26.7%)	2.38	0.17–33.51	0.520
Septic shock	7 (4.3%)	2 (1.2%)	34.94	4.19–291.53	0.001

* Median values of our study sample. Abbreviations: BSI (bloodstream infection), CDI (*Clostridioides difficile* infection), CI (confidence interval), CRP (C-reactive protein), ESCMID (European Society of Clinical Microbiology and Infectious Diseases), LTCF (long-term care facility), OR (Odds ratio), PNA (pneumonia), WBC (white blood cell).

## Data Availability

The original contributions presented in this study are included in the article/Supplementary Materials. Further inquiries can be directed to the corresponding authors.

## Supplementary Materials

The following supporting information can be downloaded at https://www.mdpi.com/article/10.3390/antibiotics15010023/s1.

## Abbreviations

The following abbreviations are used in this manuscript:

ABSSSI	Acute bacterial skin and skin structure infections
BSI	Bloodstream infection
CA-CDI	Community-acquired *Clostridioides difficile* infection
CCI	Charlson comorbidity index
CD	*Clostridioides difficile*
CDI	*Clostridioides difficile* infection
CI	Confidence interval
CKD	Chronic kidney disease
CNSI	Central nervous system infection
CRP	C-reactive protein
ECDC	European centre for disease prevention and control
ESCMID	European society of clinical microbiology and infectious diseases
GDH	Glutamate dehydrogenase
HA-CDI	Healthcare-associated *Clostridioides difficile* infection
HAI	Healthcare-associated infection
IAI	Intra-abdominal infection
IBD	Inflammatory bowel disease
iCDI	Initial CDI
ID	Infectious disease
IDSA	Infectious Diseases Society of America
IL	Interleukin
IM	Intramuscolar
IQR	Interquartile range
IV	Intravenous
LOS	Length of hospital stay
LTCF	Long-term care facility
NAAT	Nucleic acid amplification test
OR	Odds ratio
pCDI	Primary CDI
PCR	Polymerase chain reaction
PCT	Procalcitonin
PNA	Pneumonia
PPI	Proton pump inhibitor
rCDI	Recurrent CDI
sCDI	Secondary CDI
SD	Standard deviation
TOX	Toxin
UTI	Urinary tract infection
WBC	White blood cell
