# Supplementary material for: Burden of Clostridioides difficile Infection and Risk Factors for Recurrences in an Italian Tertiary Care University Hospital: A Prospective Observational Study
Source: Antibiotics (Basel). 2025 Dec 25;15(1):23. doi: 10.3390/antibiotics15010023 (PMC12837728; doi:10.3390/antibiotics15010023)
Supplement: Supplementary file 1 [file antibiotics-15-00023-s001.zip › antibiotics-3995094-supplementary.pdf]

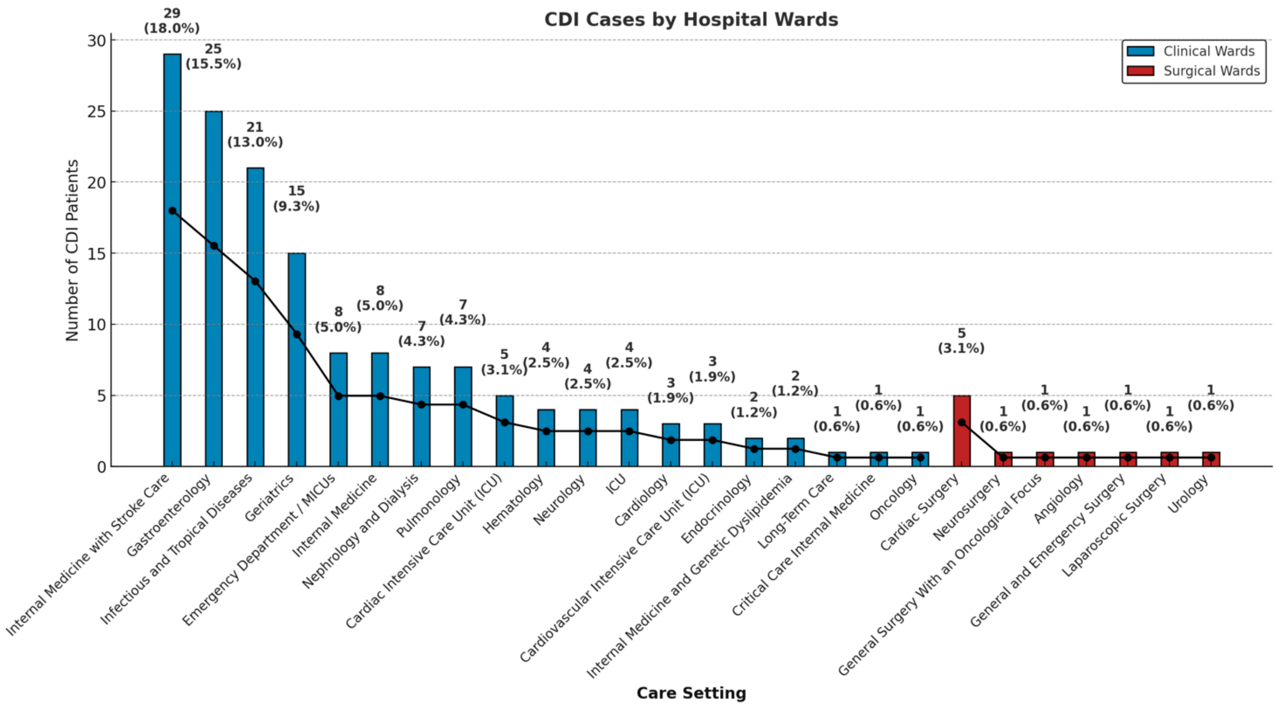

Figure S1. Ward-level distribution of CDI cases.

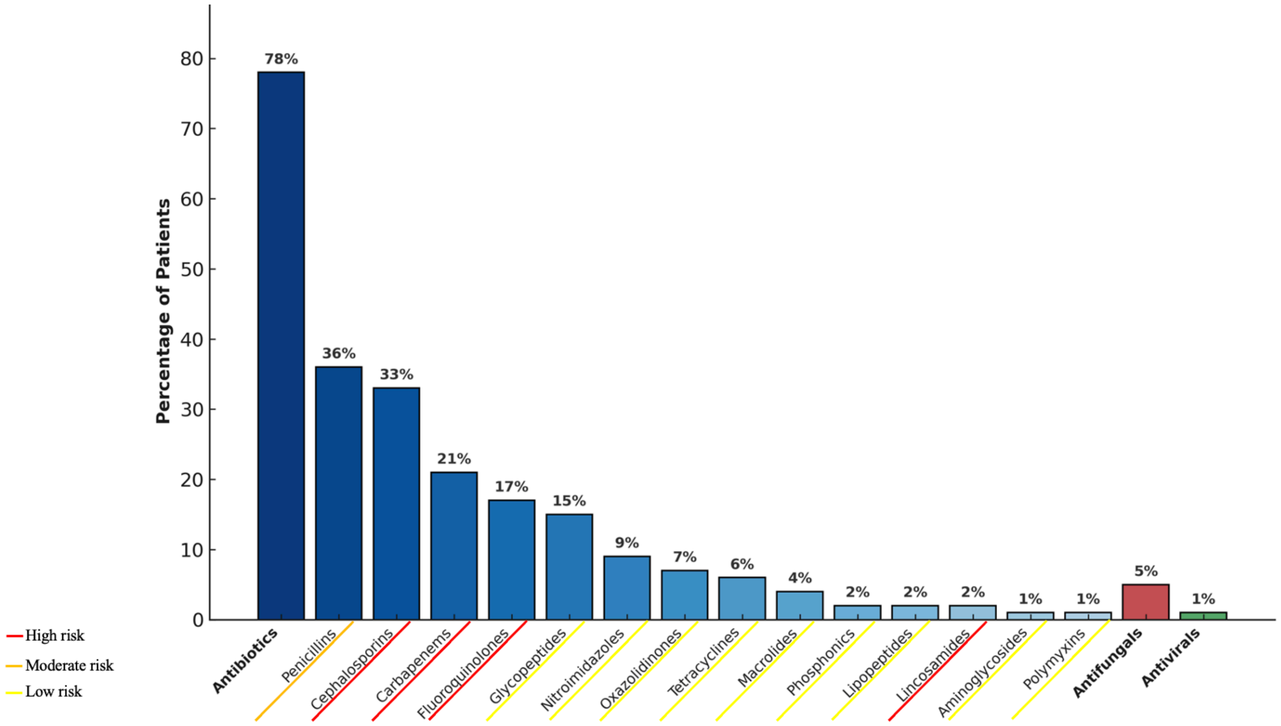

Figure S2. Distribution of antibiotics, antivirals, and antifungals administered for contextual infection, including CDI-associated risk classification.

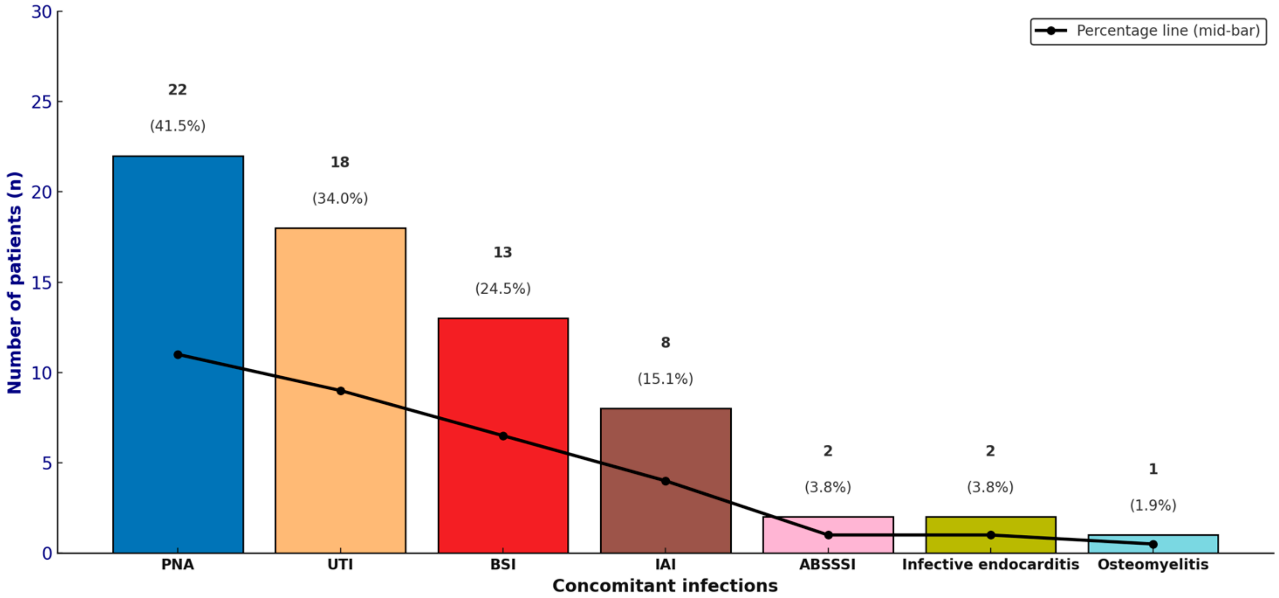

Figure S3. Distribution of concomitant infections among 53 of 126 patients (42.1%) who continued antibiotic therapy during the CDI episode. Fifteen patients (28.3%) presented with ≥2 concurrent infections. CDI treatment in this subgroup included oral vancomycin in 34 patients (64.1%), rectal vancomycin in 1 (1.9%), and fidaxomicin in 18 (34.0%). Bezlotoxumab and metronidazole were co-administered in 2 cases each.

Abbreviations: BSI (bloodstream infection), PNA (pneumonia), UTI (urinary tract infection), IAI (intra-abdominal infection), ABSSSI (acute bacterial skin and skin structure infection).
